# PRAISE-H study exploring real-world challenges faced by reproductive-age patients with rheumatoid arthritis in Japan

**DOI:** 10.3389/fmed.2026.1773628

**Published:** 2026-03-09

**Authors:** Chinatsu Takai, Izumi Fujioka, Mikako Goto

**Affiliations:** Japan Drug Information Institute in Pregnancy, Integrated Center for Women's Health, National Center for Child Health and Development, Tokyo, Japan

**Keywords:** biologic DMARDs, financial toxicity, patient-reported outcomes, pregnancy registry, real-world data, reproductive-age patients, rheumatoid arthritis

## Abstract

We explored the design and preliminary findings of the Pregnancy and Rheumatoid Arthritis Registry (PRAISE-H), a Japan-wide prospective registry of reproductive-age patients with rheumatoid arthritis (RA) that captures challenges in daily life and complements large database research. PRAISE-H is a nationwide multicentre prospective observational study in which physician assessments and patient-reported outcomes are collected biannually via REDCap over 5 years, with dedicated follow-up during pregnancy and up to 12 months postpartum. As of November 2024, 100 patients (90 women and 10 men) had been registered, and 77 patients (70 women and seven men) were under follow-up. Preliminary analyses showed that 62% of women expressed current or future pregnancy intention. In addition, substantial financial toxicity was observed, particularly among patients receiving biologic disease-modifying antirheumatic drugs (bDMARDs). By emphasizing patients' experiences in everyday environments, PRAISE-H provides insights beyond the scope of conventional database studies. These findings may inform clinical guidelines and health policy initiatives aimed at improving the management of RA in women of childbearing age. Although bDMARDs are increasingly considered viable treatment options during pregnancy, their associated financial consequences warrant further investigation.

## 1 Introduction

Rheumatoid arthritis (RA) is one of the most prevalent inflammatory rheumatic diseases (IRD) affecting women of childbearing age. Planning pregnancy in women with IRD involves multiple considerations across the entire reproductive trajectory, from the preconception period through pregnancy and after childbirth, including the impact of disease and disease activity, medication use, and maternal psychological health (1). In particular, a prolonged time to pregnancy has been consistently reported in women with RA (2, 3). In Japan, a recent nationwide claims-based study further demonstrated that the time to delivery was significantly prolonged in women with RA compared with those without the disease (4). Several factors have been proposed to underlie these findings, including inflammatory cytokines that may affect embryo implantation, the effects of antirheumatic medications (5–7), and insufficient shared decision-making (8). Among these, the contribution of high disease activity has been highlighted (5), underscoring the importance of appropriate disease control during the pregnancy planning phase.

Although methotrexate remains a key drug for the treatment of RA, it must be avoided because of its association with increased rates of teratogenicity and miscarriage (9). In this context, reports demonstrating that tumor necrosis factor (TNF) inhibitors enable low disease activity maintenance during pregnancy (10) have led to the view that biologic disease-modifying antirheumatic drugs (bDMARDs) can be beneficial for women who wish to conceive, from the pregnancy planning stage through pregnancy. This accumulating evidence is reflected in the 2024 EULAR recommendations, endorsing selected bDMARDs during pregnancy based on maternal and fetal safety data (11). Nevertheless, in real-world clinical practice in Japan, avoidance or delay of treatment for financial reasons remains a frequent challenge. The high cost of biologics highlights the importance of considering social determinants of health such as employment and economic situations when managing reproductive-age RA.

To adequately understand real-world RA management, observational cohorts with diverse clinical and social backgrounds are warranted to complement the big data approaches (12). Although the use of birth registries has recently increased (13, 14), such data sources lack detailed clinical information, including disease activity, treatment decision-making, and patient-reported outcomes. Additionally, despite international efforts, no pregnancy registry for RA currently exists in Japan (15, 16).

To bridge this gap, the Pregnancy and Rheumatoid Arthritis Registry in Hokkaido (PRAISE-H) was established as a Japan-originated prospective registry integrating clinical data with patient participation indicators across the preconception, pregnancy, postpartum, and child-bearing periods. This report primarily describes the establishment and design of the PRAISE-H registry while providing an exploratory analysis of early data to demonstrate the study's feasibility.

## 2 Materials and methods

### 2.1 Study design and participants

The PRAISE-H is a Japanese multicentre, prospective, observational registry. The 2010 American College of Rheumatology/European League Against Rheumatism classification criteria were used as a reference (17); nevertheless, each attending physician determined the final diagnosis. We aimed to focus on patients who developed RA during their reproductive years, particularly female patients in the preconception and perinatal periods, and accurately capture the treatment course from the first therapy initiation. However, for a prolonged treatment course, initial or interim treatment information is often unavailable when patients are transferred between institutions. Therefore, to maximize the possibility of obtaining a treatment history with sufficient accuracy, we limited the inclusion period to within 3 years of diagnosis for female patients.

Male patients were also included. Nonetheless, because RA is less prevalent in this group, the duration since diagnosis was not restricted. The inclusion criteria were as follows: (1) women aged 20–40 years with physician-diagnosed RA within 3 years of diagnosis; (2) men aged 20–40 years with physician-diagnosed RA, regardless of disease duration; and (3) ability to provide consent and complete electronic surveys. Written informed consent was obtained at the time of enrolment. Participant enrolment began in February 2021 and is planned to continue until the end of March 2028.

### 2.2 Follow-up

**Figure 1:**
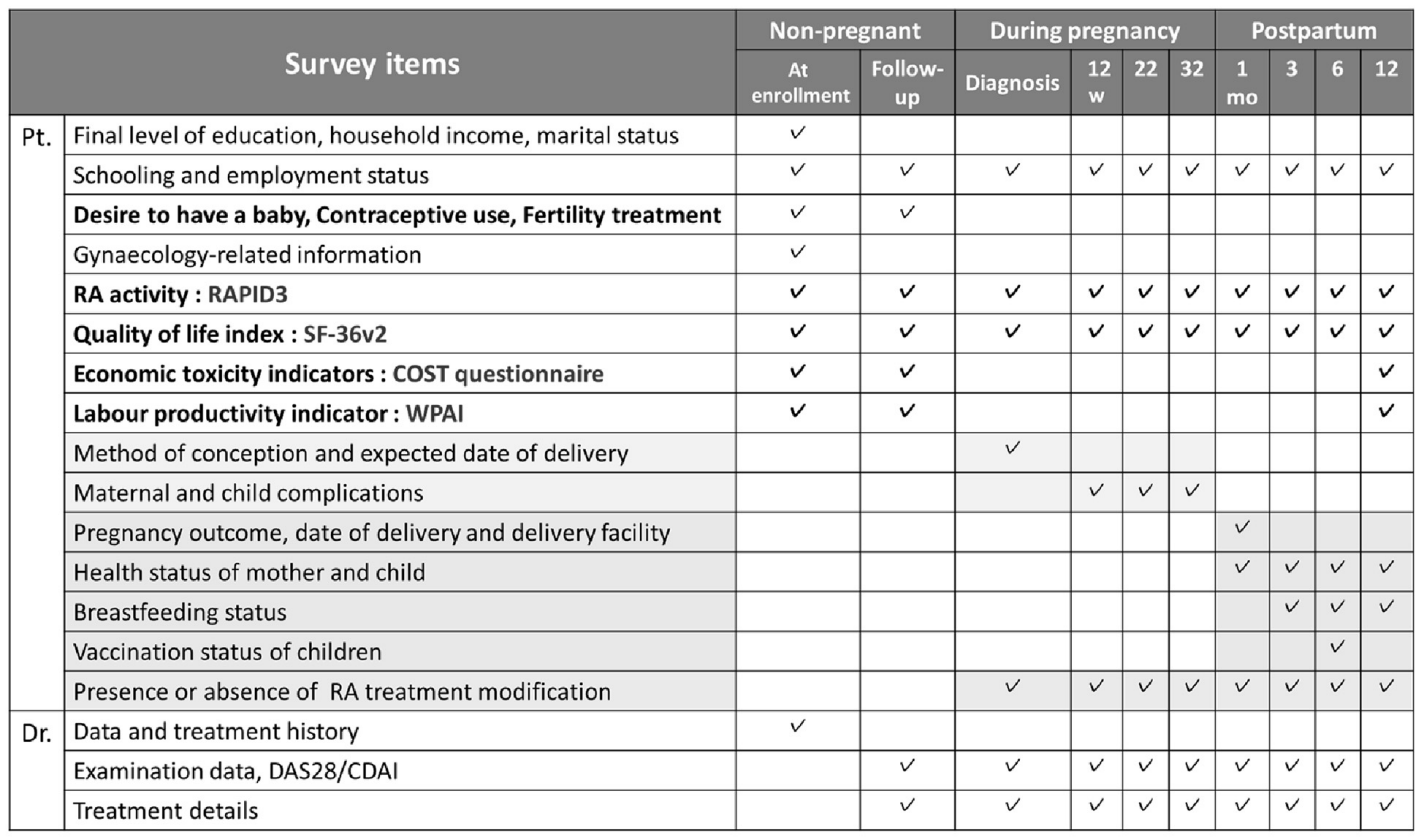
Data collection and measurements for female patients RAPID3: composite scores of physical function, pain, and global health. Remission (≤3), Low (3.1–6), Moderate (6.1–12), High (>12); SF-36v2: measures 8 QoL domains, summarized into Physical and Mental scores. Higher scores indicated better health (mean = 50, SD = 10); COST: an 11-item tool on financial toxicity (0–44). Lower scores = greater financial burden; WPAI: six items on absenteeism, presenteeism, and work/activity impairment. Scores 0%−100%, higher = greater impairment. CDAI, Clinical Disease Activity Index; COST, Comprehensive Score for Financial Toxicity; DAS28, Disease Activity Score in 28 joints. RA, Rheumatoid Arthritis; RAPID3, Routine Assessment of Patient Index Data 3; SF-36v2, 36-Item Short Form Health Survey version 2; WPAI, Work Productivity and Activity Impairment questionnaire.

Participants are followed for up to 5 years by sending emails to both patients and physicians, and their responses are collected through the REDCap system. Study data were collected and managed using REDCap (Research Electronic Data Capture) (18, 19), a secure, web-based software platform designed to support data capture for research studies, hosted at the National Center for Child Health and Development. Non-pregnant participants (men and women) are assessed every 6 months. Women who are pregnant at baseline or become pregnant during follow-up are assessed at pregnancy confirmation, at 12, 22, and 32 weeks, and postpartum at 1, 3, 6, and 12 months, after which they resume 6-monthly follow-up (Figure 1).

Physicians report diagnosis date, treatments (drug, dose, start/stop, reason), comorbidities, disease activity (physician global, Clinical Disease Activity Index, Disease Activity Score in 28 joints), and routine RA laboratories (e.g., rheumatoid factor, anti-cyclic citrullinated peptide, C-reactive protein, erythrocyte sedimentation rate, matrix metalloproteinase-3, autoantibodies). Moreover, patients report demographics, education, employment (including weekly hours), lifestyle factors, fertility intentions, contraception, and—for women—gynaecologic/thyroid history and prior pregnancy outcomes. Patient-reported outcomes (PROs) include Routine Assessment of Patient Index Data three (RAPID3), Short Form-36 version 2 (SF-36v2) (Japanese), Comprehensive Score for Financial Toxicity (COST), and Work Productivity and Activity Impairment (WPAI) at prespecified time points. RAPID3 captures function, pain, and global health (20); SF-36v2 assesses multidimensional health-related quality of life (21); COST measures financial toxicity, defined as the subjective financial distress and objective economic burden associated with medical care (22, 23); and WPAI evaluates absenteeism, presenteeism, and activity impairment (24). The pregnancy modules capture conception methods, maternal disease activity and treatment, obstetric course, delivery outcomes, neonatal status, breastfeeding, and vaccination. Additionally, the presence of and satisfaction with counseling provided by healthcare professionals regarding pregnancy, childbirth, breastfeeding, and childcare were collected for women at 1 year postpartum and for male patients whose partners had given birth.

### 2.3 Statistical analysis

Descriptive statistics were used to summarize the data. Continuous variables are presented as medians with interquartile ranges or ranges. Categorical variables are expressed as frequencies and percentages. For comparisons of COST scores between groups, JMP version 12.2.0 (SAS Institute Inc., Cary, NC, United States) was used; *p* < 0.05 was considered statistically significant.

### 2.4 Ethics and data management

The study protocol was approved by the participating institutional review boards (2019–168). Identifying information is stored separately in a central monitoring system.

## 3 Results

### 3.1 Patient characteristics at enrolment

As of November 2024, 33 facilities participated, of which 12 had actually implemented registration. There were 100 registered patients (90 women and 10 men). Due to the limited number of male participants, this preliminary analysis focused on the 90 female patients. Of these, eight were pregnant at the time of registration.

Table 1 displays the demographic characteristics of the 90 women.

**Table 1:** Demographic and clinical characteristics of female patients at enrolment (*n* = 90).

**Characteristics**	**Value**
Age, years, median (IQR)	33 (29–37)
BMI, kg/m^2^, median (IQR)	20.8 (19.5–23.6)
**Smoking history**, ***n*** **(%)**	
Never	72 (80.0)
Past	14 (15.6)
Current	4 (4.4)
**Partner status**, ***n*** **(%)**	
Yes	56 (62.2)
No	31 (34.4)
Prefer not to answer	3 (3.3)
**Serological features**, ***n*** **(%)**	
Anti-CCP antibody positive	60 (66.7)
Rheumatoid factor positive	58 (64.4)
Anti-SS-A antibody positive	21 (23.3)
CRP, mg/dL, mean ± SD	0.73 ± 1.15
**Pregnancy history**, ***n*** **(%)**	
Nulliparous (none)	55 (61.1)
Primiparous (once)	16 (17.8)
Multiparous (>2)	17 (18.9)
**Gynecologic disorders**, ***n*** **(%)**	
Uterine fibroids	4 (4.4)
Endometriosis	4 (4.4)
Cervical anomalies	3 (3.3)
Ovarian cysts	2 (2.2)
Other	5 (5.6)
**Cervical cancer screening**, ***n*** **(%)**	
Yes	65 (72.2)
No	25 (27.8)

Anti-CCP, anti-cyclic citrullinated peptide; Anti-SS-A, anti-Sjögren's-syndrome-related antigen A; BMI, body mass index; CRP, C-reactive protein; IQR, interquartile range.

### 3.2 Future childbearing intentions

**Figure 2:**
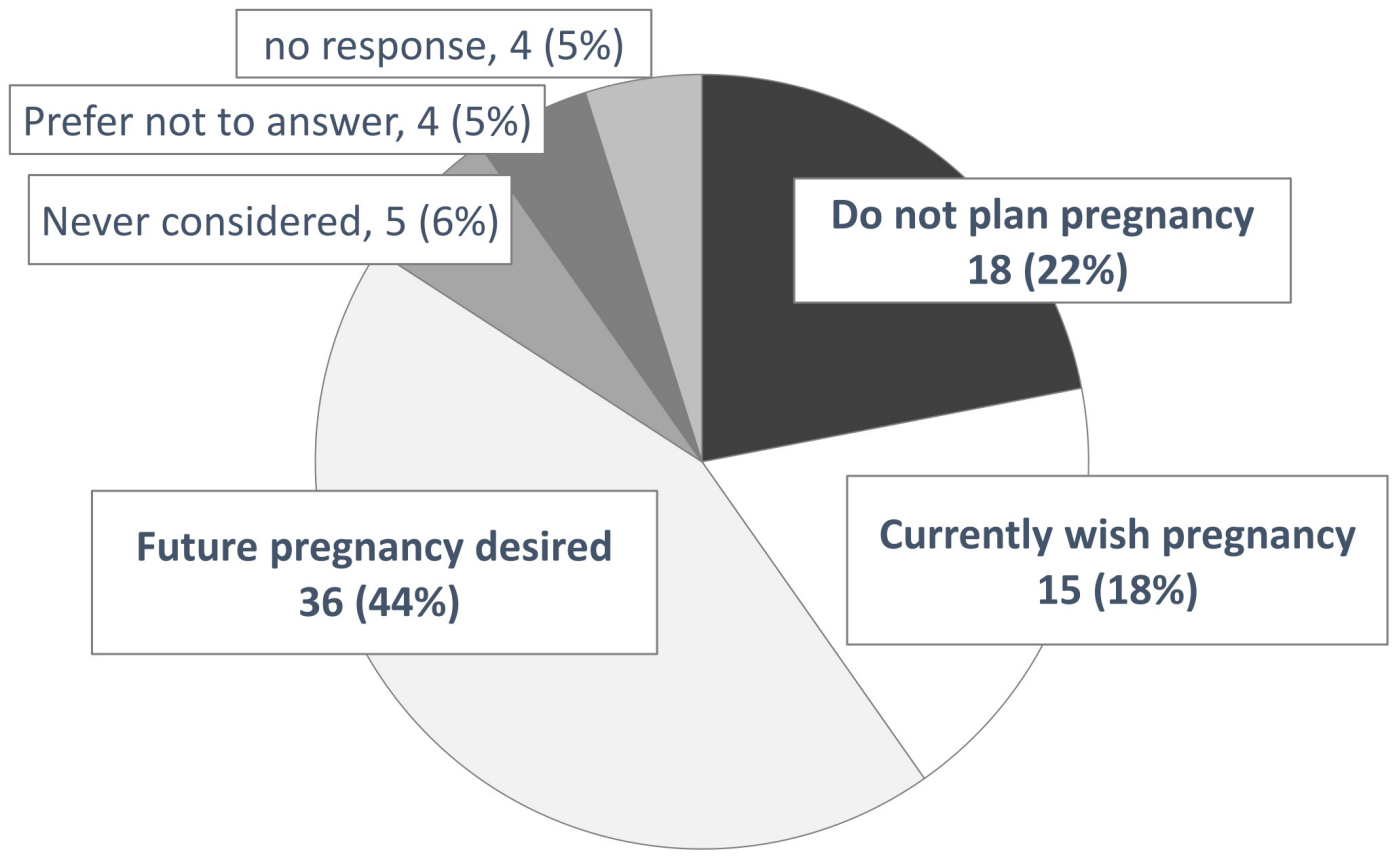
Pregnancy and childbearing intentions at registry enrolment among female patients.

Figure 2 displays the results regarding pregnancy intentions at registration for 82 women, excluding eight who were already pregnant at registration. The answers were selected from the following six options: (1) “I do not plan to become pregnant”; (2) “I currently wish to become pregnant”; (3) “I have no specific plans at present but wish to consider pregnancy in the future”; (4) “I have not given it any particular thought”; (5) “Other”; and (6) “I do not wish to answer this question”. Eighteen women (22%) selected option (1). When asked about their reasons (multiple responses allowed), non-RA-related factors were most frequently reported; however, a subset of participants cited concerns related to RA symptoms and antirheumatic treatment, indicating potentially modifiable factors through medical intervention (Table 2).

**Table 2:** Reasons for selecting (1) ‘I do not plan to become pregnant' (multiple responses allowed; *n* = 18).

**Reasons**	**“Due to RA-related symptoms”**	**“Concerns about balancing RA with pregnancy, childbirth, or childcare”**	**“Because of using medications contraindicated for pregnancy due to RA”**	**“Reasons unrelated to RA”**	**Prefer not to answer**
Number	2	4	4	15	1

RA, rheumatoid arthritis.

Conversely, 15 (18%) and 36 (44%) women selected options (2) and (3), respectively, indicating that 62% of the participants intended to become pregnant.

**Figure 3:**
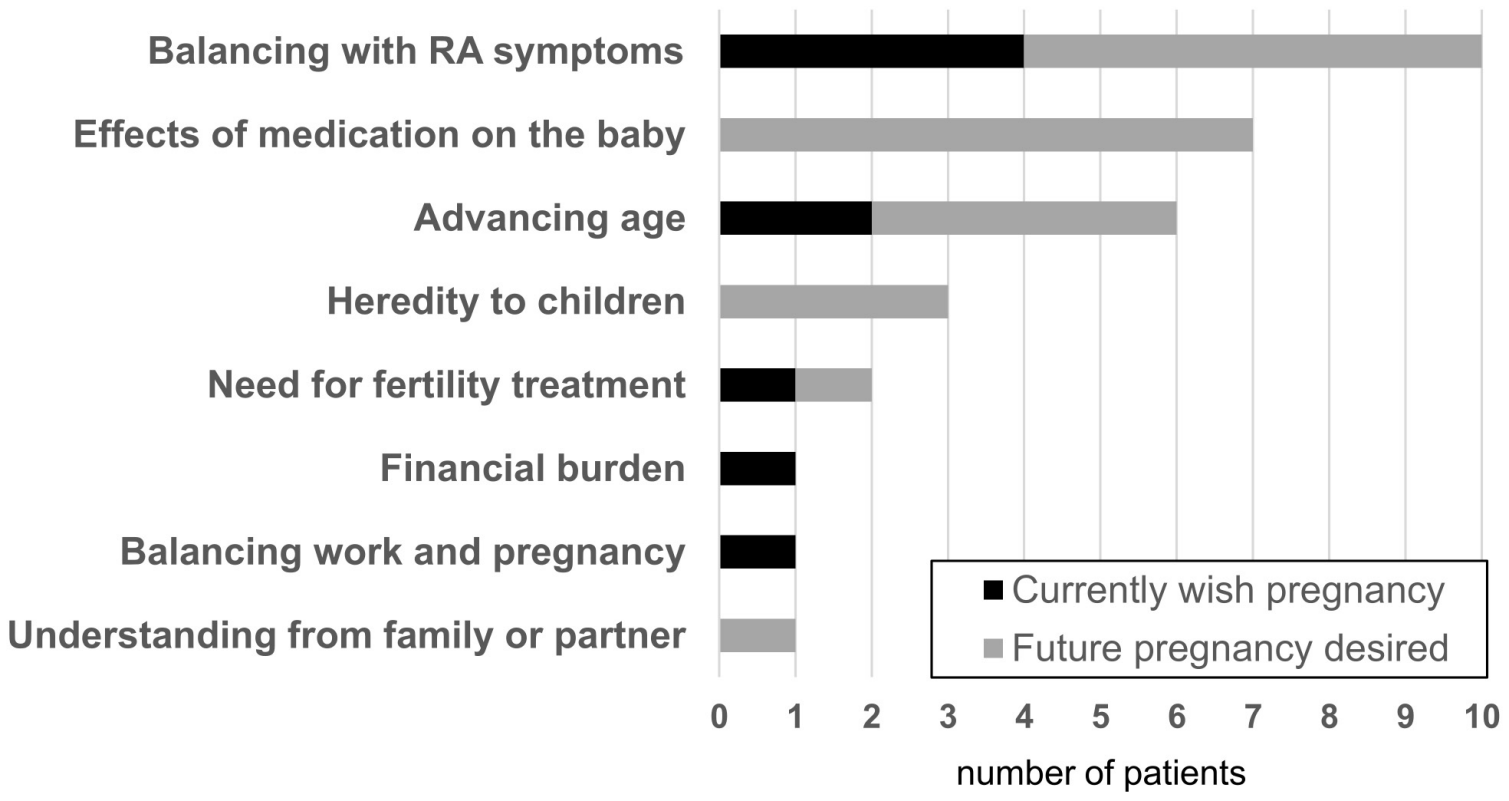
Concerns regarding future pregnancy among women who wish to become pregnant. RA, rheumatoid arthritis.

Subsequently, these participants were asked the open-ended question, “Do you have any concerns when considering a future pregnancy*?*” A summary of the free-text responses is presented in Figure 3. The most frequently reported concerns were RA-related issues, including balancing RA symptoms and the effects of medications on infants. Non-RA-related concerns included advancing age, need for fertility treatment, financial burden, and balancing work and pregnancy. These patterns were similar between women who currently wished to become pregnant and those who desired a future pregnancy.

Table 3 displays the proportions of methotrexate (MTX) and bDMARDs use and the remission achievement rate according to the desire for pregnancy. MTX use was higher among those without the desire, and over half did not achieve remission. Even among those with the desire, the non-remission rate remained at 33.3%.

**Table 3:** The proportion of MTX and bDMARD use and the remission achievement rate according to pregnancy desire.

**Clinical variables**	**Currently wish pregnancy (*n* = 15)**	**Do not plan pregnancy (*n* = 18)**
MTX use current/past/never (*n*, %)	4 (26.7%)/4 (26.7%)/7 (46.7%)	14 (77.8%)/0 (0%)/4 (22.2%)
bDMARDs current/past/never (*n*, %)	7 (46.7%)/0 (0%)/8 (53.3%)	7 (38.9%)/1 (5.6%)/10 (55.6%)
**RA remission status**		
Sustained/relapsed/not achieved (*n*, %)	7 (46.7%)/3 (20.0%)/5 (33.3%)	7 (38.9%)/1 (5.6%)/10 (55.6%)

bDMARDs, biologic disease-modifying antirheumatic drugs; MTX, methotrexate; RA, rheumatoid arthritis.

### 3.3 Financial toxicity

**Figure 4:**
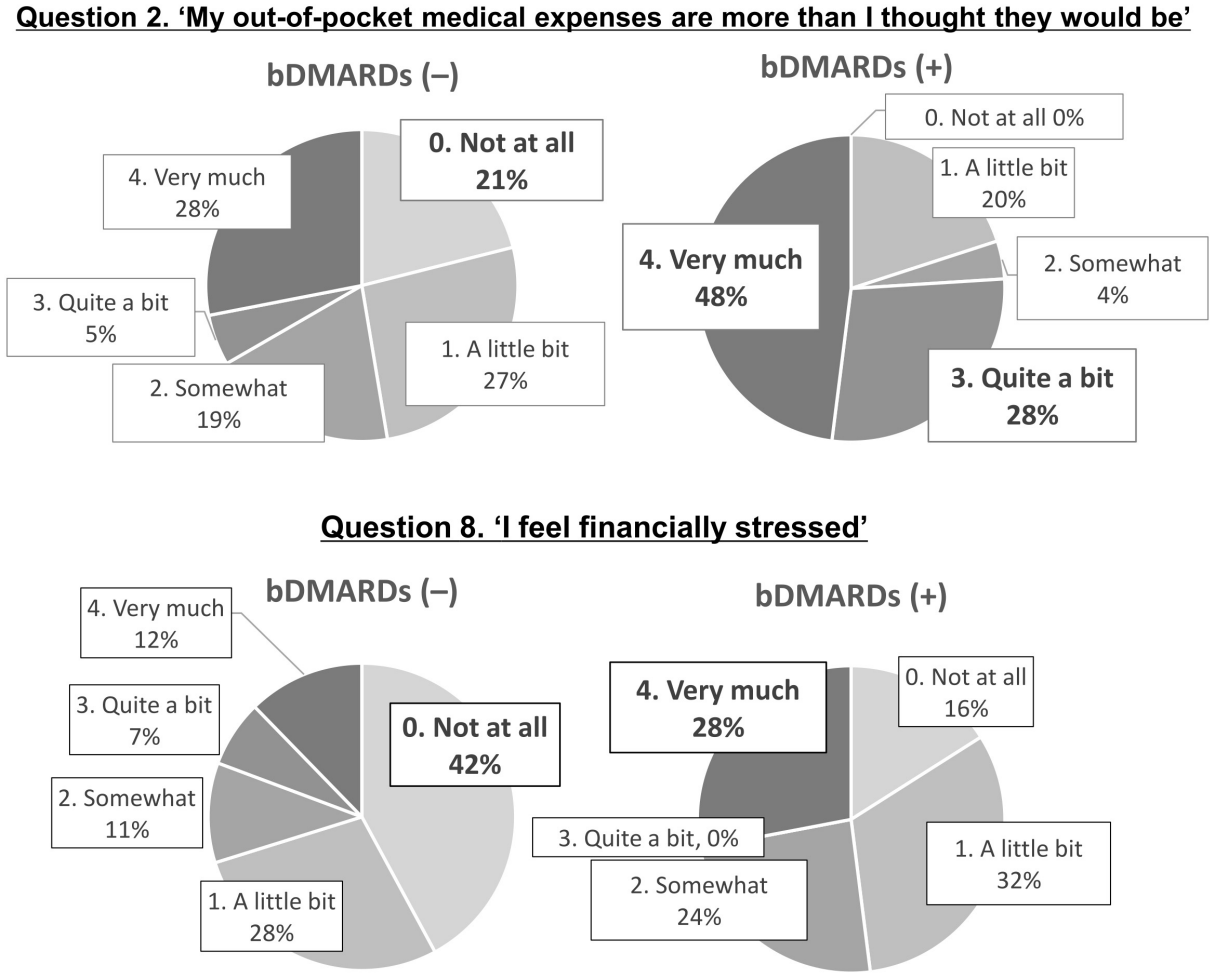
Comparison of responses to COST Questions **(2)** and **(8)** between bDMARDs-exposed and -unexposed groups. bDMARDs, biologic disease-modifying antirheumatic drugs.

Classification by COST score (0–44, lower scores indicating greater economic toxicity) demonstrated severe (0–15), moderate (16–29), and minimal to none (30–44) toxicity in 32%, 35%, and 33% of the patients, respectively. Notably, for question 8) “I feel financially stressed”, a marked difference in perceived burden was observed between those using bDMARDs and those who did not (Figure 4).

We compared financial toxicity between patients with and without bDMARD treatment using the COST score. The median COST score was 16 (IQR, 10–24) and 22 (IQR, 14–28) in the bDMARD-exposed and -unexposed group, respectively, indicating significantly greater toxicity in the exposed group (Mann–Whitney *U* test, *p* = 0.022). Following previous studies, COST scores were categorized into four grades: ≥26 = no impact (Grade 0), 14–25 = mild impact (Grade 1), 1–13 = moderate impact (Grade 2), and 0 = severe impact (Grade 3). The proportion of patients with financial toxicity (Grade ≥1) was 80.0% and 57.9% in the bDMARD-exposed and -unexposed groups, respectively (Table 4). Although this difference was not statistically significant, financial toxicity prevalence tended to be higher in the exposed group.

**Table 4:** Comparison of COST scores and financial toxicity grades between bDMARD-exposed and -unexposed groups.

**bDMARD exposure**	**COST score median (range)**	**Grade 0**	**Grade 1**	**Grade 2**	**Grade 3**	**Financial toxicity (+) (grade > = 1)**
		**Score** > = **26 (no impact)**	**Score 14–25 (mild)**	**Score 1–13 (moderate)**	**Score 0 (severe)**	
bDMARDs (+) *n* = 25	16 [10–24]	5 (20%)	9 (36%)	11 (44%)	0	20 (80%)
bDMARDs (–) *n* = 57	22 [14–28]	24 (42.1%)	19 (33.3%)	14 (24.6%)	0	33 (57.9%)

bDMARDs, biologic disease-modifying antirheumatic drugs; COST, Comprehensive Score for Financial Toxicity.

## 4 Discussion

Disease registries provide valuable resources for evaluating physicians' prescribing patterns and treatment trend changes, particularly when maintained over many years. This registry is a Japanese multicentre prospective platform that longitudinally tracks RA patients of reproductive age from the non-pregnant phase through the perinatal and postpartum periods, integrating clinical indicators with PROs. This report primarily describes the establishment and design of the PRAISE-H registry. It presents an exploratory analysis of early data to demonstrate its feasibility, with a focus on women in the preconception period.

### 4.1 Patient background and women's healthcare issues

The background characteristics of the 90 enrolled female patients were broadly representative of the general RA population, suggesting that the registry functioned as intended. Importantly, this analysis identified women's healthcare issues beyond RA itself, including low body mass index (BMI) and limited access to gynecological care. These findings highlight the unmet healthcare needs that may influence reproductive decision-making independent of RA.

### 4.2 Pregnancy intention and disease activity

Delayed pregnancy onset has been reported in women with RA (7), with high disease activity cited as a contributing factor (1). In a longitudinal observational study in the USA, conducted in an era without options for using bDMARDs during pregnancy, 55% of women with RA had fewer children than desired; this outcome might be attributable to infertility, which was reported by 42% of these women (5). Here, 61% of participants had no history of pregnancy. The prospective design of PRAISE-H will clarify how background factors, disease activity, and treatment status influence subsequent pregnancy outcomes.

Reproductive healthcare management of RA in women of childbearing age has evolved in recent decades. While the updated 2024 EULAR recommendations continue to contraindicate MTX before and during pregnancy owing to teratogenicity, a key shift is a more permissive stance toward bDMARDs, supported by accumulating safety data (11, 25). Specifically, TNF inhibitors can be used continuously during the second trimester with minimal effects.

Here, although over half of the women expressed a desire for pregnancy, the remission rate was low among those without such a desire, suggesting that not achieving remission may prevent them from developing a desire to conceive. Notably, even among women who explicitly reported no desire for pregnancy, some cited concerns related to RA symptoms or treatment, suggesting that timely pre-pregnancy counseling may help address modifiable barriers. Moreover, although high disease activity may discourage pregnancy planning, this association is likely bidirectional. For women who are not actively planning conception, physicians may be less likely to intensify therapy to achieve strict remission, and patients may prioritize other life goals. Longitudinal data from PRAISE-H will be critical for disentangling this relationship, rather than assuming a single causal pathway.

### 4.3 Treatment status and financial aspects of pregnancy planning

Here, the high utilization of MTX and bDMARDs among women without a desire for pregnancy suggests that pregnancy attempts may be restricted by high disease activity or difficulty in discontinuing MTX. Although bDMARDs may provide better disease control and are increasingly considered compatible with pregnancy, their high cost remains a major barrier (26). Accordingly, patients receiving bDMARDs reported a substantially higher financial burden compared to those who did not. Importantly, while biologic DMARDs are increasingly used in clinical practice during pregnancy, the supporting evidence varies among agents, underscoring the importance of individualized counseling rather than uniform assumptions about acceptability.

### 4.4 Value of a registry starting before pregnancy

To address limitations of conventional registries, which often enroll patients only after pregnancy confirmation (27), PRAISE-H conducts longitudinal assessments from the non-pregnant phase, including contraceptive use, fertility evaluation, and pregnancy intention changes. A key future challenge is to identify effective interventions when pregnancy desires emerge. Additionally, this registry captures delayed decision-making (28) and information needs through open-ended responses, enabling evaluation of approaches to information provision. Furthermore, background characteristics highlighted broader healthcare issues for women in Japan, including low BMI and low cervical cancer screening rates, which may represent opportunities for intervention before pregnancy planning.

This registry uses PROs (RAPID3, SF-36v2, COST, WPAI). Although some COST data have been presented, bDMARD use during pregnancy and lactation may have increased the perceived financial burden. Japan has unique medical cost subsidies; however, these are rarely applied to patients with RA. These data may provide a basis for future policy recommendations in Japan, where declining birth rates are a significant issue. Case accumulation is required to clarify the actual situation.

Although not presented in the current analysis, 12 participants had given birth at the time of manuscript preparation, and further accumulation of pregnancy and delivery outcomes was anticipated.

### 4.5 Limitations

A significant limitation of this study is the small number of cases, which reflects the early phase of the registry and our focus on data precision and real-world representation. By limiting inclusion to patients within 3 years of diagnosis, we aimed to minimize recall bias regarding initial treatments. In addition, recruitment spans a diverse range of clinical settings, including both academic institutions and private clinics. While this approach may slow initial recruitment compared with large-scale database studies, it provides a more granular and representative view of RA management in Japan. In particular, the number of male patients was limited in the current analysis, despite the growing interest in male reproductive health. Nevertheless, focusing on male patients whose partners have given birth represents a novel and valuable approach for exploring an area that has been largely overlooked in both domestic and international research.

## 5 Conclusions

PRAISE-H is among the first Japanese registries to center on reproductive-age RA with systematic PRO measurements and planned longitudinal pregnancy follow-up. Early indicators suggest modifiable barriers, including remission gaps, gynaecologic access, and financial toxicity, which extend beyond inflammation control, with implications for counseling, treatment selection, and health policy.

## Data Availability

The original contributions presented in the study are included in the article/supplementary material, further inquiries can be directed to the corresponding author.
